# Author Correction: Spatial proteomics reveals secretory pathway disturbances caused by neuropathy-associated TECPR2

**DOI:** 10.1038/s41467-023-43990-w

**Published:** 2023-12-14

**Authors:** Karsten Nalbach, Martina Schifferer, Debjani Bhattacharya, Hung Ho-Xuan, Wei Chou Tseng, Luis A. Williams, Alexandra Stolz, Stefan F. Lichtenthaler, Zvulun Elazar, Christian Behrends

**Affiliations:** 1 Munich Cluster for Systems Neurology (SyNergy), Medical Faculty, Ludwig-Maximilians-University München, Munich, Germany; 2 German Center for Neurodegenerative Diseases (DZNE), Munich, Germany; 3 Munich Cluster for Systems Neurology (SyNergy), Munich, Germany; 4 Buchmann Institute for Molecular Life Sciences and Institute of Biochemistry II, Faculty of Medicine, Goethe University Frankfurt, Frankfurt, Germany; 5 Q-State Biosciences, 179 Sidney Street, Cambridge, MA 02139 USA; 6 Neuroproteomics, School of Medicine, Klinikum rechts der Isar, Technical University of Munich, Munich, Germany; 7 Departments of Biomolecular Sciences, The Weizmann Institute of Science, Rehovot, Israel

**Keywords:** Mechanisms of disease, Secretion, Proteomics

Correction to: *Nature Communications* 10.1038/s41467-023-36553-6, published online 16 February 2023

This Article contains an error in the name of the author Wei Chou Tseng, which is incorrectly given as Wei Tseng. This has been corrected in both the PDF and HTML versions of the Article.

In the original version of this Article, the affiliation details for Buchmann Institute for Molecular Life Sciences and Institute of Biochemistry II, Faculty of Medicine, Goethe University Frankfurt, Frankfurt, Germany were incorrectly given as Buchmann Institute for Molecular Life Sciences, Goethe University Frankfurt, Frankfurt, Germany. This has now been corrected in both the PDF and HTML versions of the Article.

The original version of this Article contained an error in the Acknowledgements which incorrectly read ‘the Collaborative Research Center SFB 1177 (ID 259130777 (C.B.)’ The correct version states ‘(C.B., A.S.)’ in place of ‘(C.B.)’. This has been corrected in both the PDF and HTML versions of the Article.

