# Supplementary material for: Spatial proteomics reveals secretory pathway disturbances caused by neuropathy-associated TECPR2
Source: Nat Commun. 2023 Feb 16;14:870. doi: 10.1038/s41467-023-36553-6 (PMC9935918; doi:10.1038/s41467-023-36553-6)
Supplement: Supplementary file 1 — Supplementary Information [file 41467_2023_36553_MOESM1_ESM.pdf]

Supplementary Information

Spatial proteomics reveals secretory pathway disturbances caused by neuropathy-associated TECPR2

Karsten Nalbach[1], Martina Schifferer[2,3], Debjani Bhattacharya[1], Hung Ho-Xuan[4], Wei Tseng[5], Luis A. Williams[5], Alexandra Stolz[4], Stefan F. Lichtenthaler[2,3,6], Zvulun Elazar[7], Christian Behrends[1,*]

[1] Munich Cluster for Systems Neurology (SyNergy), Medical Faculty, Ludwig-Maximilians-University München, Munich, Germany

[2] German Center for Neurodegenerative Diseases (DZNE), Munich, Germany

[3] Munich Cluster for Systems Neurology (SyNergy), Munich, Germany

[4] Buchmann Institute for Molecular Life Sciences, Goethe University Frankfurt, Frankfurt, Germany

[5] Q-State Biosciences, 179 Sidney Street, Cambridge, MA 02139, USA

[6] Neuroproteomics, School of Medicine, Klinikum rechts der Isar, Technical University of Munich, Munich, Germany

[7] Departments of Biomolecular Sciences, The Weizmann Institute of Science, Rehovot, Israel

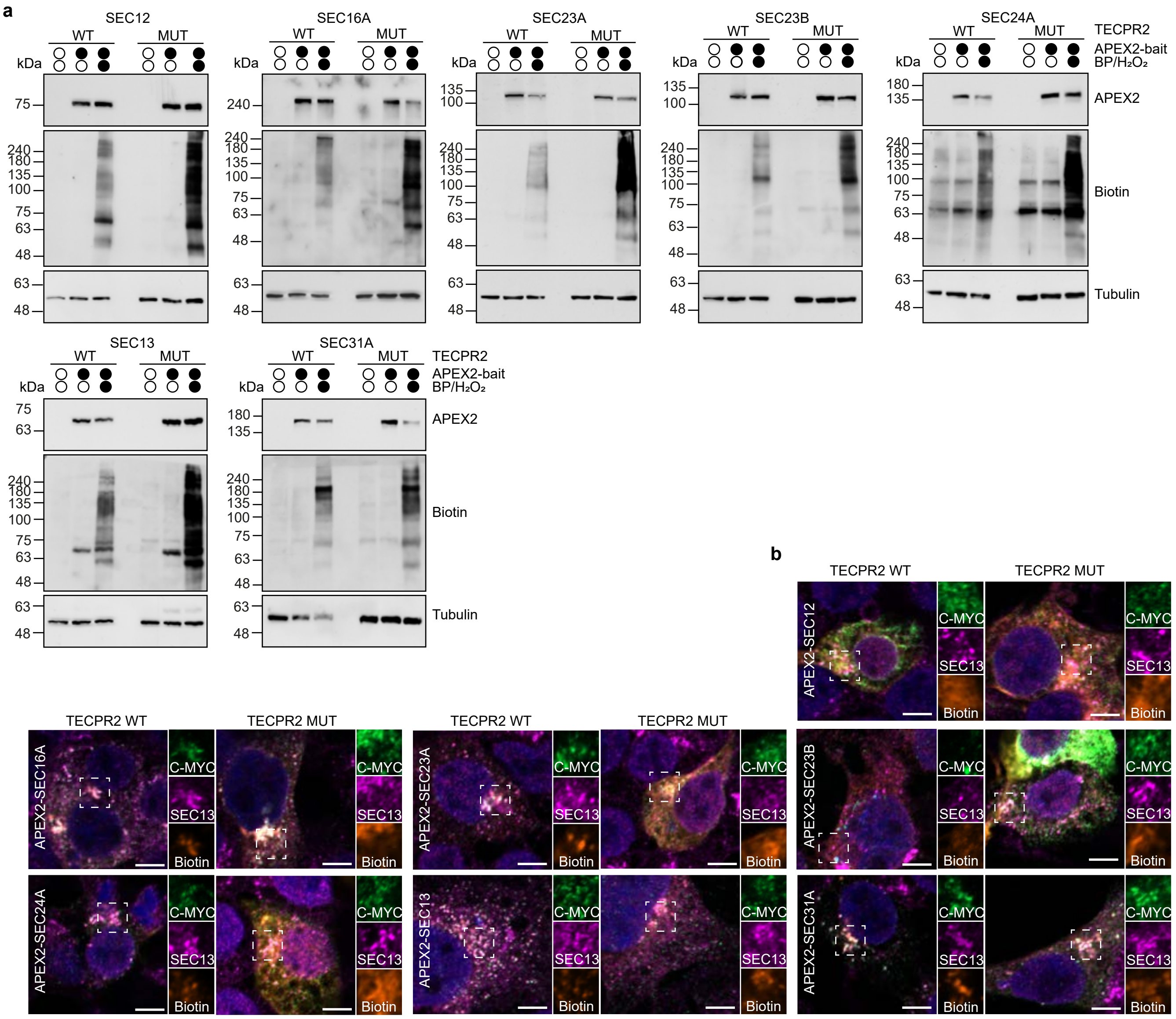
a
SEC12
SEC16A
SEC23A
SEC23B
SEC24A
WT
MUT
TECPR2
APEX2-bait
BP/H₂O₂
kDa
APEX2
Biotin
Tubulin
240
180
135
100
75
63
48
SEC13
SEC31A
b
TECPR2 WT
TECPR2 MUT
APEX2-SEC12
APEX2-SEC23B
APEX2-SEC31A
APEX2-SEC16A
APEX2-SEC24A
APEX2-SEC23A
APEX2-SEC13
C-MYC
SEC13
Biotin

Supplementary Figure 1: **APEX2-ERES cell line evaluation.**

a, b TECPR2 WT and MUT cells expressing APEX2-SEC12, -SEC16A, -SEC23A, -SEC23B, -SEC24A, -SEC13 or -SEC31A were differentially treated with biotin-phenol (BP) and H_2O_2 to induce biotinylation followed by lysis and immunoblotting (**a**) or fixation and immunolabeling (**b**) with indicated antibodies. Insets show magnification of boxed areas. Scale bars 10 µm. Source data are provided in Source Data file.

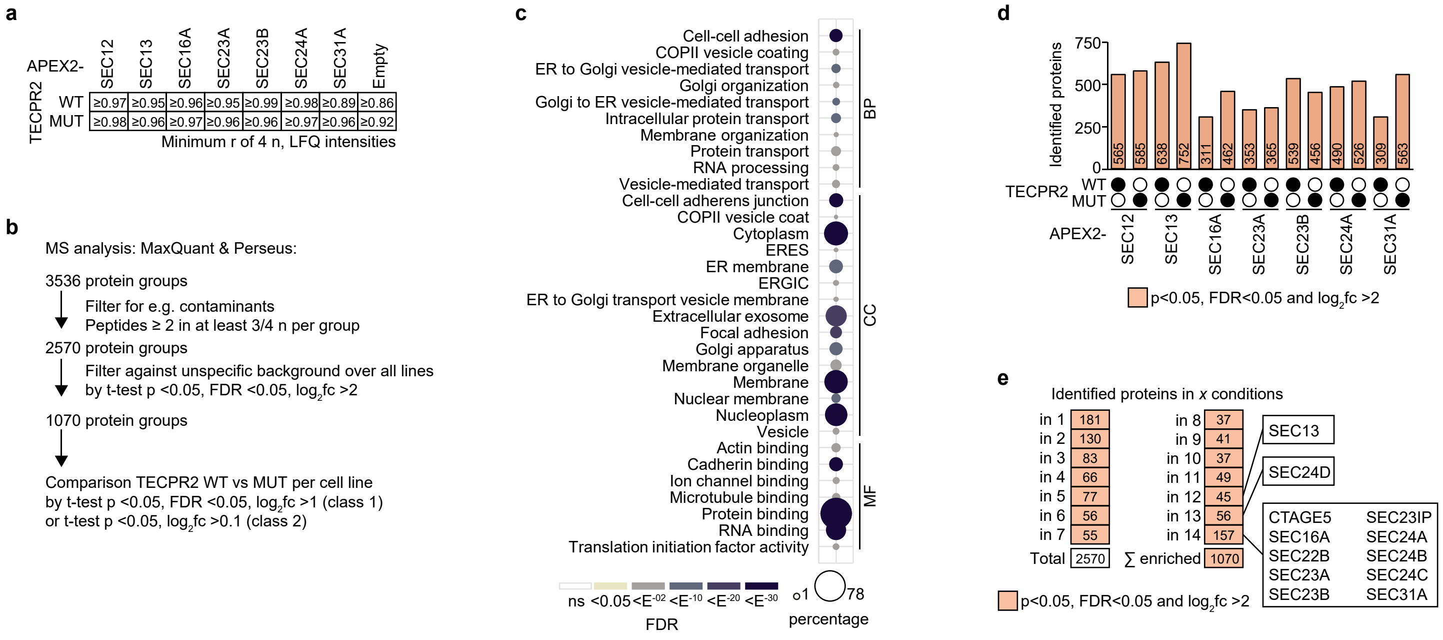
a
APEX2-
SEC12
SEC13
SEC16A
SEC23A
SEC23B
SEC24A
SEC31A
Empty
TECPR2
WT ≥0.97 ≥0.95 ≥0.96 ≥0.95 ≥0.99 ≥0.98 ≥0.89 ≥0.86
MUT ≥0.98 ≥0.96 ≥0.97 ≥0.96 ≥0.96 ≥0.97 ≥0.96 ≥0.92
Minimum r of 4 n, LFQ intensities
b
MS analysis: MaxQuant & Perseus:
3536 protein groups
Filter for e.g. contaminants
Peptides ≥ 2 in at least 3/4 n per group
2570 protein groups
Filter against unspecific background over all lines
by t-test p <0.05, FDR <0.05, log2fc >2
1070 protein groups
Comparison TECPR2 WT vs MUT per cell line
by t-test p <0.05, FDR <0.05, log2fc >1 (class 1)
or t-test p <0.05, log2fc >0.1 (class 2)
c
Cell-cell adhesion
COPII vesicle coating
ER to Golgi vesicle-mediated transport
Golgi organization
Golgi to ER vesicle-mediated transport
Intracellular protein transport
Membrane organization
Protein transport
RNA processing
Vesicle-mediated transport
Cell-cell adherens junction
COPII vesicle coat
Cytoplasm
ERES
ER membrane
ERGIC
ER to Golgi transport vesicle membrane
Extracellular exosome
Focal adhesion
Golgi apparatus
Membrane organelle
Membrane
Nuclear membrane
Nucleoplasm
Vesicle
Actin binding
Cadherin binding
Ion channel binding
Microtubule binding
Protein binding
RNA binding
Translation initiation factor activity
BP
CC
MF
ns <0.05 <E-02 <E-10 <E-20 <E-30
FDR
1 78
percentage
d
Identified proteins
750
500
250
0
565 585 638 752 311 462 353 365 539 456 490 526 309 563
TECPR2 WT
MUT
APEX2-
SEC12
SEC13
SEC16A
SEC23A
SEC23B
SEC24A
SEC31A
p<0.05, FDR<0.05 and log2fc >2
e
Identified proteins in x conditions
in 1 181
in 2 130
in 3 83
in 4 66
in 5 77
in 6 56
in 7 55
Total 2570
in 8 37
in 9 41
in 10 37
in 11 49
in 12 45
in 13 56
in 14 157
∑ enriched 1070
SEC13
SEC24D
CTAGE5
SEC16A
SEC22B
SEC23A
SEC23B
SEC23IP
SEC24A
SEC24B
SEC24C
SEC31A
p<0.05, FDR<0.05 and log2fc >2

Supplementary Figure 2: **ERES proximity profiling.**

a Pearson correlation of four biological replicates based on LFQ intensities of identified and quantified proteins. **b** Schematic representation of proteomic data filtering and statistical analysis. **c** GO term enrichment of all proximity proteins that were significantly enriched compared to non-biotinylated control cells. Dot size correlates to the number of proteins, dot color to term enrichment (FDR) (two-sided t-test, n=4 independent experiments). **d** Number of identified proteins per APEX2 chimera in TECPR2 WT and MUT cells that were enriched over unspecific background (two-sided t-test, n=4 independent experiments). **e** Comparative analysis of identified proteins across different APEX2 chimeras in TECPR2 WT and MUT cells (two-sided t-test, n=4 independent experiments). Source data are provided in Source Data file.

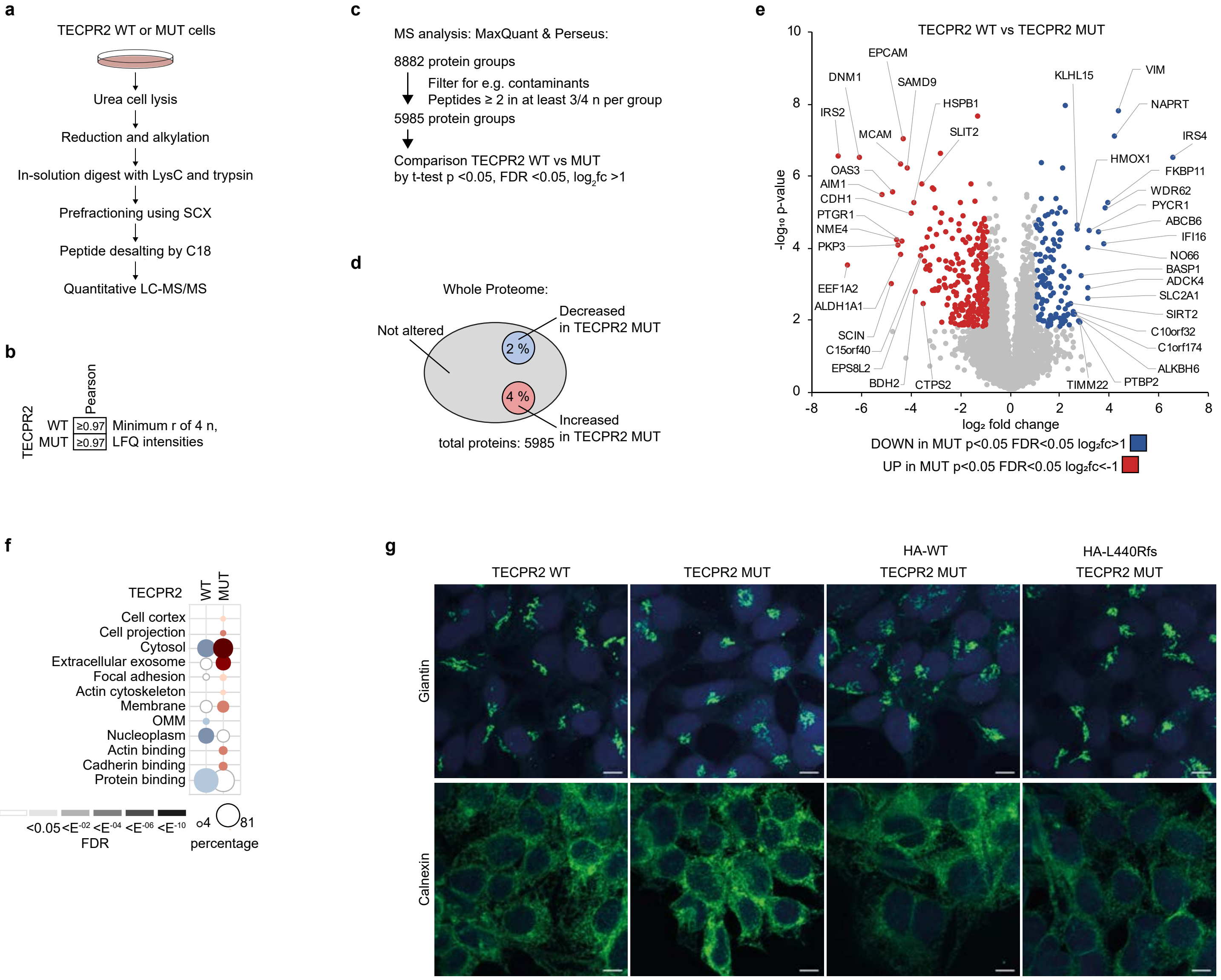
a
TECPR2 WT or MUT cells
Urea cell lysis
Reduction and alkylation
In-solution digest with LysC and trypsin
Prefractioning using SCX
Peptide desalting by C18
Quantitative LC-MS/MS
b
Pearson
TECPR2
WT ≥0.97 Minimum r of 4 n,
MUT ≥0.97 LFQ intensities
c
MS analysis: MaxQuant & Perseus:
8882 protein groups
Filter for e.g. contaminants
Peptides ≥ 2 in at least 3/4 n per group
5985 protein groups
Comparison TECPR2 WT vs MUT
by t-test p <0.05, FDR <0.05, log2fc >1
d
Whole Proteome:
Not altered
Decreased in TECPR2 MUT
2 %
4 %
Increased in TECPR2 MUT
total proteins: 5985
e
TECPR2 WT vs TECPR2 MUT
-log10 p-value
log2 fold change
EPCAM
DNM1
SAMD9
IRS2
HSPB1
MCAM
SLIT2
OAS3
AIM1
CDH1
PTGR1
NME4
PKP3
EEF1A2
ALDH1A1
SCIN
C15orf40
EPS8L2
BDH2
CTPS2
KLHL15
VIM
NAPRT
IRS4
HMOX1
FKBP11
WDR62
PYCR1
ABCB6
IFI16
NO66
BASP1
ADCK4
SLC2A1
SIRT2
C10orf32
C1orf174
ALKBH6
PTBP2
TIMM22
DOWN in MUT p<0.05 FDR<0.05 log2fc>1
UP in MUT p<0.05 FDR<0.05 log2fc<-1
f
TECPR2
WT
MUT
Cell cortex
Cell projection
Cytosol
Extracellular exosome
Focal adhesion
Actin cytoskeleton
Membrane
OMM
Nucleoplasm
Actin binding
Cadherin binding
Protein binding
<0.05 <E-02 <E-04 <E-06 <E-10
FDR
4
81
percentage
g
TECPR2 WT
TECPR2 MUT
HA-WT TECPR2 WT
HA-L440Rfs TECPR2 MUT
Giantin
Calnexin

Supplementary Figure 3: **Global protein abundance profiling.**

a Whole proteome workflow. **b** Pearson correlation of 4 biological replicates based on LFQ intensities of identified proteins. **c** Schematic representation of whole proteome data filtering and statistical processing. **d** Venn graph-like summary of proteins decreased (blue), increased (red) or unchanged (grey) upon TECPR2 MUT compared to TECPR2 WT. **e** Volcano plot of the total proteome of TECPR2 WT compared to TECPR2 MUT cells. Proteins decreased in TECPR2 MUT cells are highlighted in blue, proteins increased in red and unchanged proteins in gray ($p < 0.05$, FDR < 0.05 and $\log_2$ fold change > 1, two-sided t-test, n=4 independent experiments), respectively. **f** GO-Term analysis of candidates with altered total protein abundance in TECPR2 MUT cells. Dot size correlates to number of proteins, dot color to term enrichment (FDR) (two-sided t-test, n=4 independent experiments). g) TECPR2 WT and MUT cells as well as TECPR2 MUT cells re-expressing TECPR2 WT and L440Rfs were fixed and immunostained with an anti-Giantin or anti-Calnexin antibody. Scale bars represent 10 µm. Source data are provided in Source Data file.

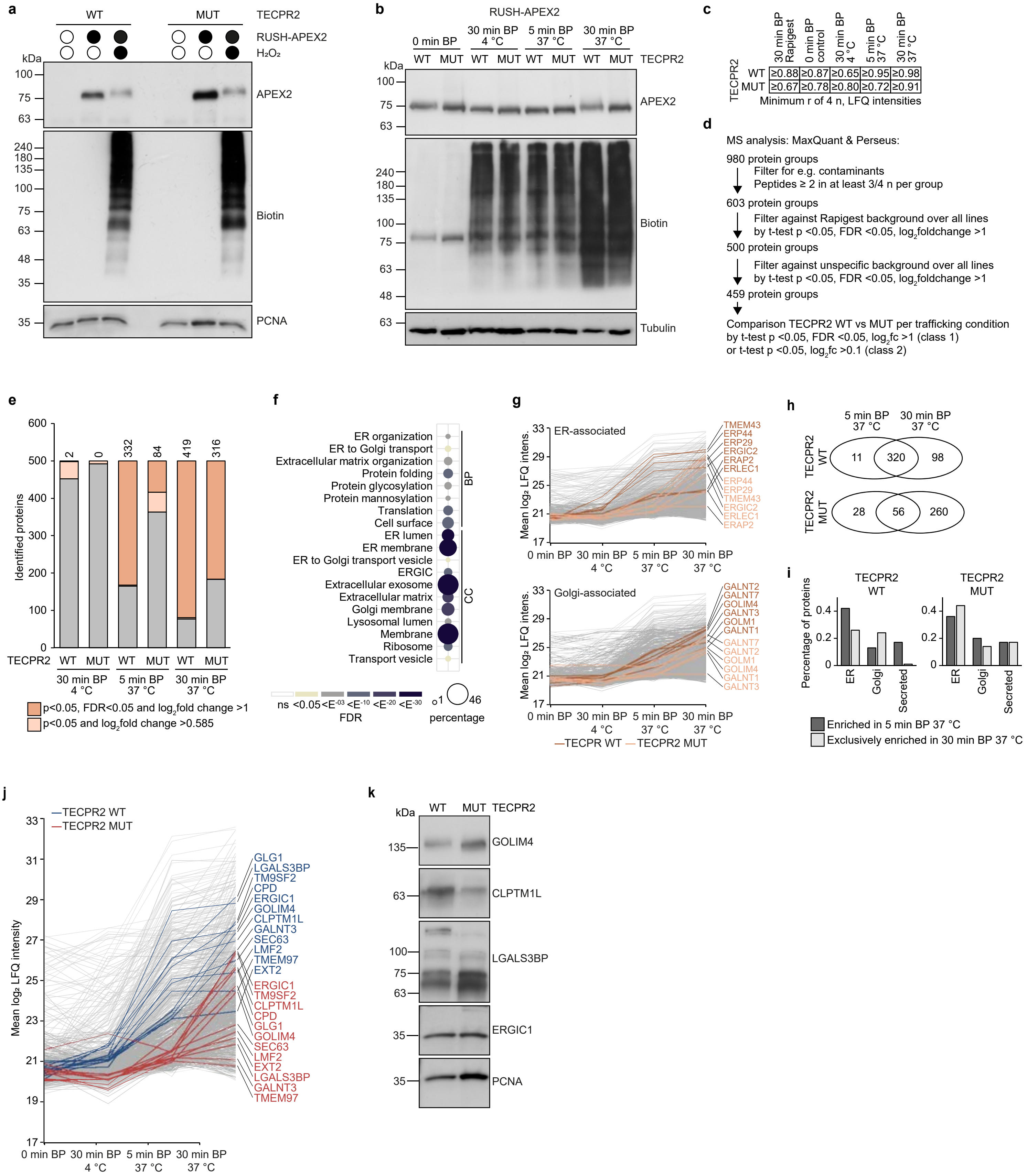

a
WT
MUT
TECPR2
RUSH-APEX2
H₂O₂
kDa
APEX2
Biotin
PCNA
b
RUSH-APEX2
0 min BP
30 min BP 4 °C
5 min BP 37 °C
30 min BP 37 °C
WT MUT WT MUT WT MUT WT MUT TECPR2
APEX2
Biotin
Tubulin
c
30 min BP Rapigest
0 min BP control
30 min BP 4 °C
5 min BP 37 °C
30 min BP 37 °C
TECPR2
WT ≥0.88 ≥0.87 ≥0.65 ≥0.95 ≥0.98
MUT ≥0.67 ≥0.78 ≥0.80 ≥0.72 ≥0.91
Minimum r of 4 n, LFQ intensities
d
MS analysis: MaxQuant & Perseus:
980 protein groups
Filter for e.g. contaminants
Peptides ≥ 2 in at least 3/4 n per group
603 protein groups
Filter against Rapigest background over all lines
by t-test p <0.05, FDR <0.05, log2foldchange >1
500 protein groups
Filter against unspecific background over all lines
by t-test p <0.05, FDR <0.05, log2foldchange >1
459 protein groups
Comparison TECPR2 WT vs MUT per trafficking condition
by t-test p <0.05, FDR <0.05, log2fc >1 (class 1)
or t-test p <0.05, log2fc >0.1 (class 2)
e
Identified proteins
2 0 332 84 419 316
TECPR2 WT MUT WT MUT WT MUT
30 min BP 4 °C
5 min BP 37 °C
30 min BP 37 °C
p<0.05, FDR<0.05 and log2fold change >1
p<0.05 and log2fold change >0.585
f
ER organization
ER to Golgi transport
Extracellular matrix organization
Protein folding
Protein glycosylation
Protein mannosylation
Translation
Cell surface
ER lumen
ER membrane
ER to Golgi transport vesicle
ERGIC
Extracellular exosome
Extracellular matrix
Golgi membrane
Lysosomal lumen
Membrane
Ribosome
Transport vesicle
BP
CC
ns <0.05 <E-03 <E-10 <E-20 <E-30
FDR
1 46
percentage
g
Mean log₂ LFQ intens.
ER-associated
TMEM43 ERP44 ERP29 ERGIC2 ERAP2 ERLEC1
ERP44 ERP29 TMEM43 ERGIC2 ERLEC1 ERAP2
Golgi-associated
GALNT2 GALNT7 GOLIM4 GALNT3 GOLM1 GALNT1
GALNT7 GALNT2 GOLM1 GOLIM4 GALNT1 GALNT3
0 min BP
30 min BP 4 °C
5 min BP 37 °C
30 min BP 37 °C
TECPR WT
TECPR2 MUT
h
5 min BP 37 °C
30 min BP 37 °C
TECPR2 WT 11 320 98
TECPR2 MUT 28 56 260
i
Percentage of proteins
TECPR2 WT
TECPR2 MUT
ER Golgi Secreted
Enriched in 5 min BP 37 °C
Exclusively enriched in 30 min BP 37 °C
j
TECPR2 WT
TECPR2 MUT
Mean log₂ LFQ intensity
GLG1 LGALS3BP TM9SF2 CPD ERGIC1 GOLIM4 CLPTM1L GALNT3 SEC63 LMF2 TMEM97 EXT2
ERGIC1 TM9SF2 CLPTM1L CPD GLG1 GOLIM4 SEC63 LMF2 EXT2 LGALS3BP GALNT3 TMEM97
0 min BP
30 min BP 4 °C
5 min BP 37 °C
30 min BP 37 °C
k
kDa
WT MUT TECPR2
GOLIM4
CLPTM1L
LGALS3BP
ERGIC1
PCNA

Supplementary Figure 4: **COPII cargo profiling by RUSH-APEX2.**

a Empty or MAN2A1-SBP-GFP (RUSH)-APEX2 expressing TECPR2 WT and MUT 293T cells grown in the presence of biotin-phenol (BP) were differentially treated with H_2O_2 and lysed for immunoblotting with anti-APEX2, -biotin and -PCNA antibodies. **b** TECPR2 WT and MUT cells expressing RUSH-APEX2 were treated for 0, 5 and 30 min with BP at 4 °C or 37 °C and pulsed with H_2O_2 prior to lysis and immunoblot analysis. **c** Pearson correlation of four biological replicates based on LFQ intensities of identified and quantified proteins. **d** Schematic representation of proximity proteomic data filtering and statistical analysis. **e** Bar graph of proteinase K protected proteins enriched after 5 min and 30 min BP treatment (orange) compared to untreated (0 min BP) control conditions (grey, unchanged) after prefiltering against proteins found in proteinase K- and RapiGest-treated background samples (two-sided t-test, n=4 independent experiments). **f** GO term enrichment of proximity proteins enriched over non-biotinylated control samples. Dot size correlates to number of proteins, dot color to term enrichment (FDR) (two-sided t-test, n=4 independent experiments). **g** Line graphs depicting mean $\log_2$ LFQ intensities of selected known ER and Golgi proteins across different trafficking conditions in TECPR2 WT (brown) or MUT (orange) cells detected by protease protection-enhanced RUSH-APEX2 profiling. Grey lines represent all proteins enriched over non-biotinylated control samples. **h** Venn graph summarizing proteins enriched after 5 min and/or 30 min trafficking at 37 °C. **i** Distribution of enriched proteins annotated as ER-associated, Golgi-associated and secreted proteins (based on GO term keywords) in TECPR2 WT and MUT cells. Proteins considered for comparison were either enriched after 5 min trafficking at 37 °C (dark grey) or exclusively enriched after 30 min trafficking at 37 °C (light grey).**j** Line graphs depicting mean $\log_2$ LFQ intensities of selected COPII cargo candidates across different trafficking conditions in TECPR2 WT (blue) and MUT (red) cells detected by protease protection-enhanced RUSH-APEX2 profiling. Grey lines represent all proteins enriched over non-biotinylated control samples. **k** Lysates from TECPR2 WT and MUT cells were analyzed by immunoblotting with indicated antibodies. PCNA served as loading control. Source data are provided in Source Data file.

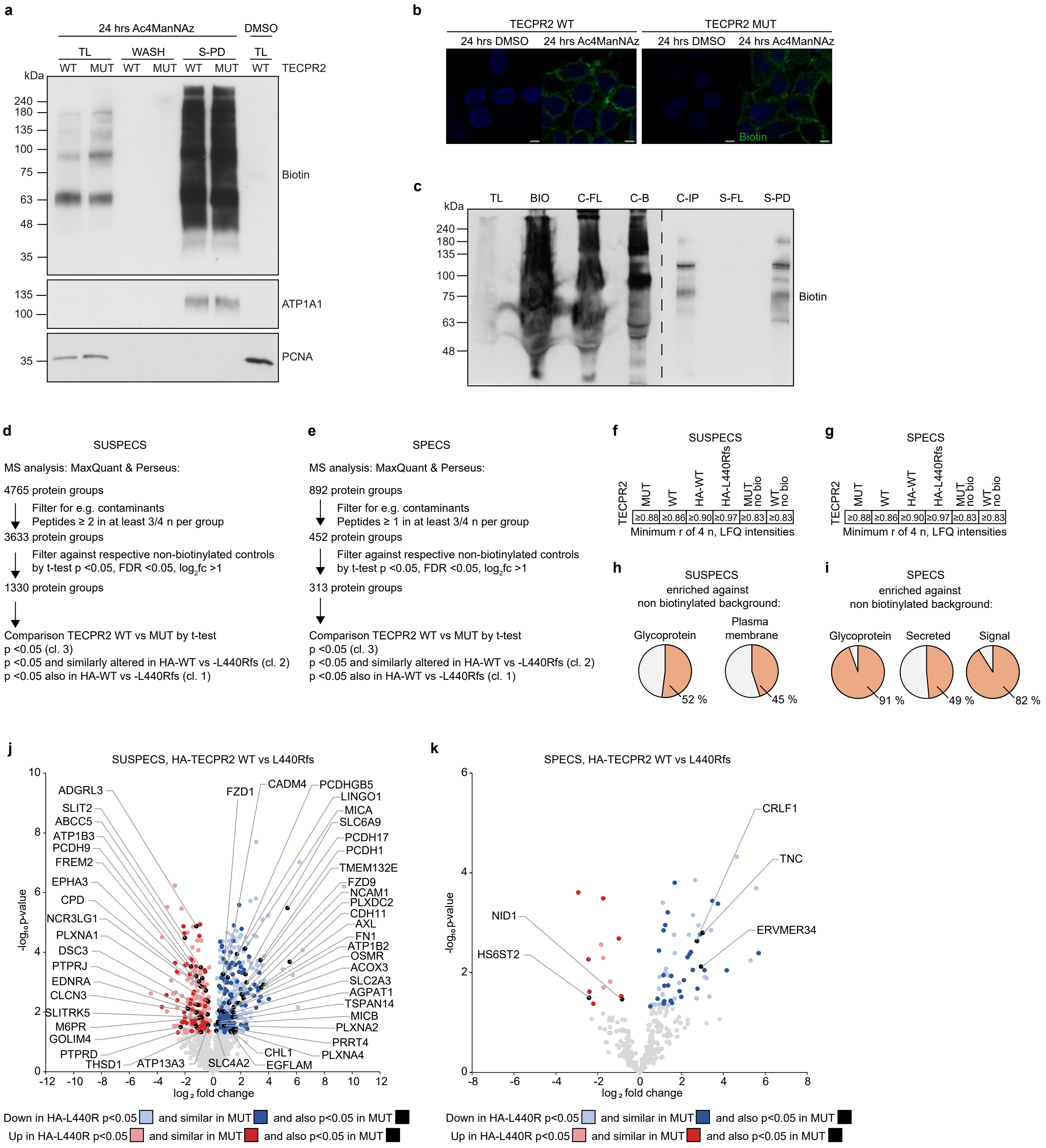

a
24 hrs Ac4ManNAz
DMSO
TL
WASH
S-PD
TL
WT
MUT
WT
MUT
WT
MUT
WT
TECPR2
kDa
240
180
135
100
75
63
48
35
Biotin
135
100
ATP1A1
35
PCNA
b
TECPR2 WT
TECPR2 MUT
24 hrs DMSO
24 hrs Ac4ManNAz
24 hrs DMSO
24 hrs Ac4ManNAz
Biotin
c
kDa
TL
BIO
C-FL
C-B
C-IP
S-FL
S-PD
240
180
135
100
75
63
48
Biotin
d
SUSPECS
MS analysis: MaxQuant & Perseus:
4765 protein groups
Filter for e.g. contaminants
Peptides ≥ 2 in at least 3/4 n per group
3633 protein groups
Filter against respective non-biotinylated controls
by t-test p <0.05, FDR <0.05, log2fc >1
1330 protein groups
Comparison TECPR2 WT vs MUT by t-test
p <0.05 (cl. 3)
p <0.05 and similarly altered in HA-WT vs -L440Rfs (cl. 2)
p <0.05 also in HA-WT vs -L440Rfs (cl. 1)
e
SPECS
MS analysis: MaxQuant & Perseus:
892 protein groups
Filter for e.g. contaminants
Peptides ≥ 1 in at least 3/4 n per group
452 protein groups
Filter against respective non-biotinylated controls
by t-test p <0.05, FDR <0.05, log2fc >1
313 protein groups
Comparison TECPR2 WT vs MUT by t-test
p <0.05 (cl. 3)
p <0.05 and similarly altered in HA-WT vs -L440Rfs (cl. 2)
p <0.05 also in HA-WT vs -L440Rfs (cl. 1)
f
SUSPECS
TECPR2
MUT
WT
HA-WT
HA-L440Rfs
MUT no bio
WT no bio
≥0.88
≥0.86
≥0.90
≥0.97
≥0.83
≥0.83
Minimum r of 4 n, LFQ intensities
g
SPECS
TECPR2
MUT
WT
HA-WT
HA-L440Rfs
MUT no bio
WT no bio
≥0.88
≥0.86
≥0.90
≥0.97
≥0.83
≥0.83
Minimum r of 4 n, LFQ intensities
h
SUSPECS
enriched against
non biotinylated background:
Glycoprotein
Plasma membrane
52 %
45 %
i
SPECS
enriched against
non biotinylated background:
Glycoprotein
Secreted
Signal
91 %
49 %
82 %
j
SUSPECS, HA-TECPR2 WT vs L440Rfs
ADGRL3
SLIT2
ABCC5
ATP1B3
PCDH9
FREM2
EPHA3
CPD
NCR3LG1
PLXNA1
DSC3
PTPRJ
EDNRA
CLCN3
SLITRK5
M6PR
GOLIM4
PTPRD
THSD1
ATP13A3
SLC4A2
FZD1
CADM4
PCDHGB5
LINGO1
MICA
SLC6A9
PCDH17
PCDH1
TMEM132E
FZD9
NCAM1
PLXDC2
CDH11
AXL
FN1
ATP1B2
OSMR
ACOX3
SLC2A3
AGPAT1
TSPAN14
MICB
PLXNA2
PRRT4
PLXNA4
CHL1
EGFLAM
-log10 p-value
log2 fold change
10
8
6
4
2
0
-12
-10
-8
-6
-4
-2
0
2
4
6
8
10
12
Down in HA-L440R p<0.05
and similar in MUT
and also p<0.05 in MUT
Up in HA-L440R p<0.05
and similar in MUT
and also p<0.05 in MUT
k
SPECS, HA-TECPR2 WT vs L440Rfs
CRLF1
TNC
ERVMER34
NID1
HS6ST2
-log10 p-value
log2 fold change
6
4
2
0
-8
-6
-4
-2
0
2
4
6
8
Down in HA-L440R p<0.05
and similar in MUT
and also p<0.05 in MUT
Up in HA-L440R p<0.05
and similar in MUT
and also p<0.05 in MUT

Supplementary Figure 5: **Cell surface proteome and secretome analysis.**

a, b TECPR2 WT and MUT 293T cells treated with DMSO or tetra-acetylated N-azidoacetylmannosamine (Ac_4-ManNAz) for 24 hrs and with sulfo-dibenzylcyclooctyne-biotin conjugate (DBCO) for 2 hrs at 4° C prior to denaturing lysis, streptavidin pulldown (S-PD) and immunoblotting (**a**) or to fixation without membrane permeabilization and immunolabeling with an anti-biotin antibody (**b**). TL, total lysate. ATP1A1 and PCNA served as loading controls. Scale bars 10 µm. **c** Immunoblot analysis of biotinylated protein enrichment from Ac4ManNAz (24 hrs)- and DBCO (ON at 4° C)-treated 293T cell derived media that were enriched for glycoproteins with concanavalin A sepharose (2 hrs at 4 °C) BIO, fraction after biotinylation; C-FL, flow-through of concanavalin A sepharose; C-B, concanavalin A pulldown; C-IP, eluate after concanavalin A; S-FL, flow-through of streptavidin sepharose; S-IP, streptavidin pulldown. **d, e** Schematic representation of SUSPECS (**d**) and SPECS (**e**) proteomic data filtering and statistical processing. **f, g** Pearson correlation of four biological replicates based on LFQ intensities of identified proteins using SUSPECS (**f**) and SPECS (**g**) proteomics. **h, i** Distribution of or proteins identified by SPECS (**h**) and SUSPECS (**i**) with glycoprotein, plasma membrane, secreted or signal peptide GO term annotations enriched against non-biotinylated background. **j, k** Volcano plot of cell surface proteome (**j**) and secretome (**k**) alterations upon TECPR2 L440Rfs expression. Significantly altered proteins (two-sided t-test, n=4 independent experiments) are grouped as class III (light color), class II (dark color) or class I (black and annotated). Source data are provided in Source Data file.

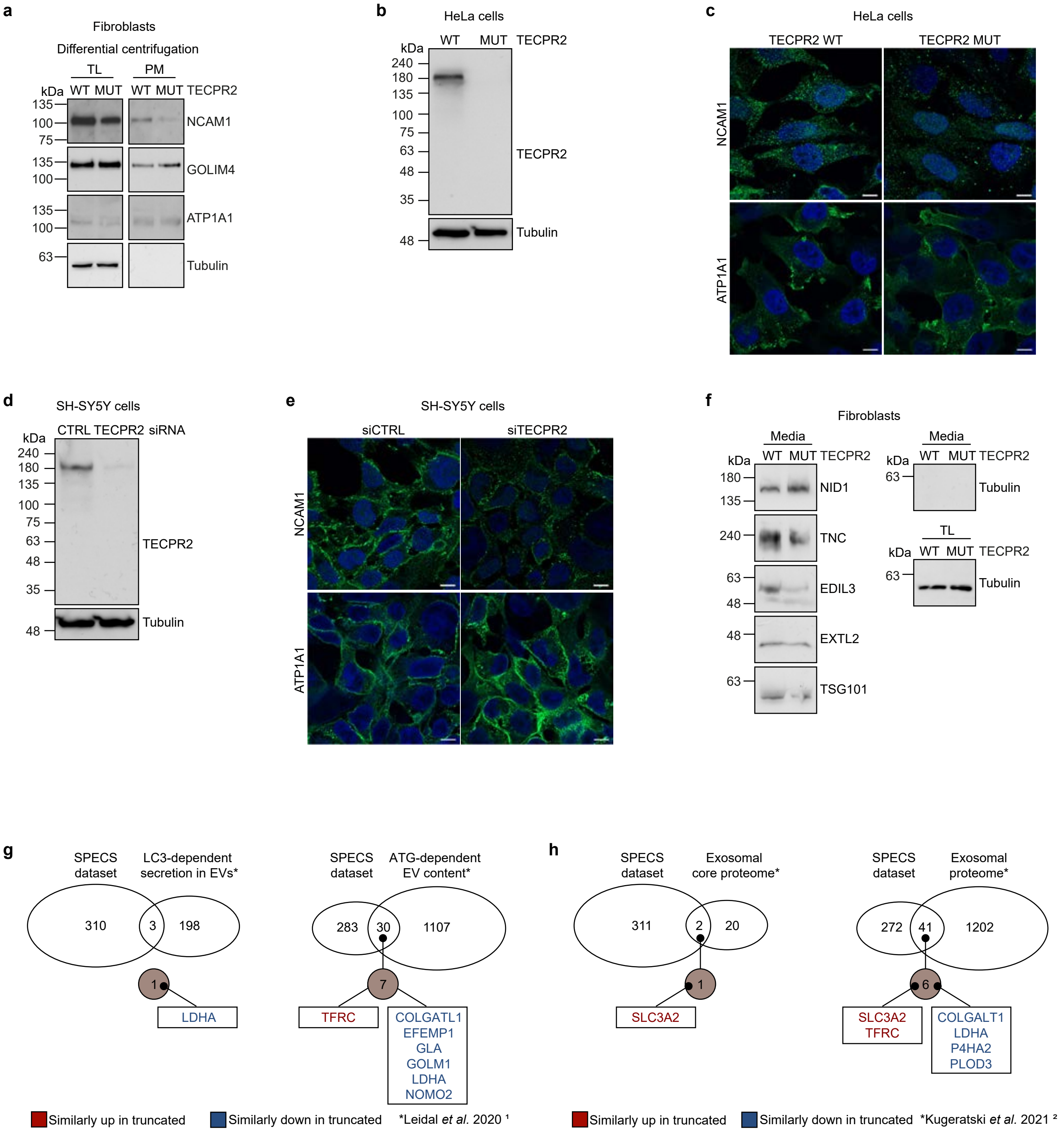
a
Fibroblasts
Differential centrifugation
TL
PM
kDa WT MUT WT MUT TECPR2
135
100
75
NCAM1
135
100
GOLIM4
135
100
ATP1A1
63
Tubulin
b
HeLa cells
WT MUT TECPR2
kDa
240
180
135
100
75
63
48
35
TECPR2
48
Tubulin
c
HeLa cells
TECPR2 WT
TECPR2 MUT
NCAM1
ATP1A1
d
SH-SY5Y cells
CTRL TECPR2 siRNA
kDa
240
180
135
100
75
63
48
35
TECPR2
48
Tubulin
e
SH-SY5Y cells
siCTRL
siTECPR2
NCAM1
ATP1A1
f
Fibroblasts
Media
kDa WT MUT TECPR2
180
135
NID1
240
TNC
63
48
EDIL3
48
EXTL2
63
TSG101
Media
kDa WT MUT TECPR2
63
Tubulin
TL
kDa WT MUT TECPR2
63
Tubulin
g
SPECS dataset
LC3-dependent secretion in EVs*
310
3
198
1
LDHA
SPECS dataset
ATG-dependent EV content*
283
30
1107
7
TFRC
COLGATL1
EFEMP1
GLA
GOLM1
LDHA
NOMO2
Similarly up in truncated
Similarly down in truncated
*Leidal et al. 2020 ¹
h
SPECS dataset
Exosomal core proteome*
311
2
20
1
SLC3A2
SPECS dataset
Exosomal proteome*
272
41
1202
6
SLC3A2
TFRC
COLGALT1
LDHA
P4HA2
PLOD3
Similarly up in truncated
Similarly down in truncated
*Kugeratski et al. 2021 ²

Supplementary Figure 6: **Validation of cell surface proteome and secretome candidates.** **a** Immunoblot of PM fractions obtained by differential centrifugation of homogenates from healthy individuum- and patient-derived fibroblasts. ATP1A1 and tubulin served as loading controls. **b, c** TECPR2 WT and MUT HeLa cells were either lysed and analyzed by immunoblotted (**b**) or fixed and immunostained with an anti-NCAM1 antibody (**c**). Scale bars represent 10 µm. **d, e** SH-SY5Y cells were reversely transfected with non-targeting siCTRL or siTECPR2 followed by lysis and immunoblotting (**d**) or fixation and immunostaining with an anti-NCAM1 antibody (**e**). Scale bars represent 10 µm. **f** Media from healthy individuum- and patient-derived fibroblasts were subjected to size-exclusion filtration and lectin-based immunoprecipitation followed by immunoblotting. EXTL2, TSG101 and tubulin served as loading controls. **g, h** Comparative analysis of all secreted proteins found in this study and those subjected to autophagy-dependent secretion [1] (**g**) or exosome secretion [2] (**h**). Brown circles indicate commonly altered proteins which are further broken down depending on their regulation in our SPECS dataset. Source data are provided in Source Data file.

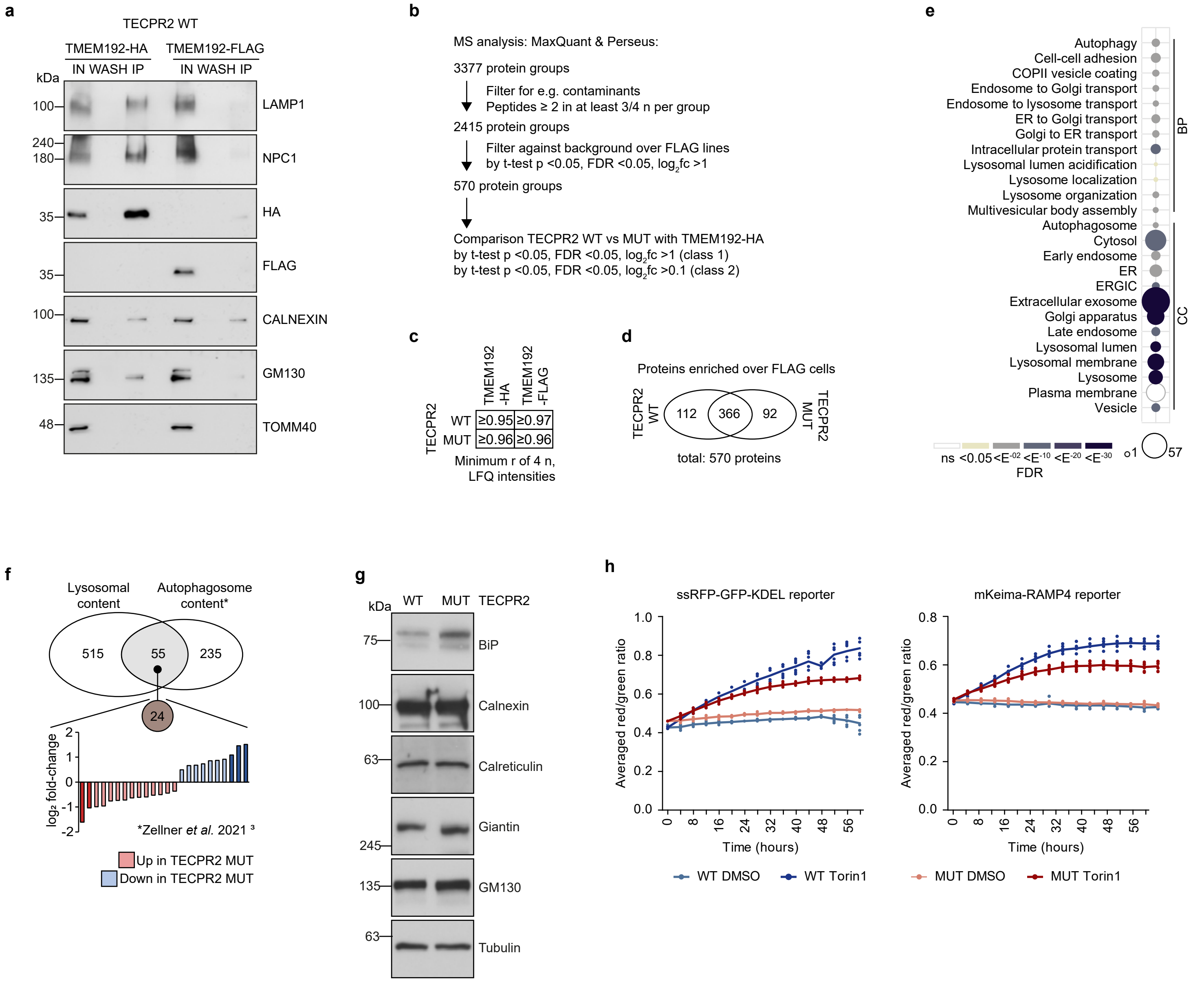
a
TECPR2 WT
TMEM192-HA
TMEM192-FLAG
IN WASH IP
IN WASH IP
kDa
100
240
180
35
35
100
135
48
LAMP1
NPC1
HA
FLAG
CALNEXIN
GM130
TOMM40
b
MS analysis: MaxQuant & Perseus:
3377 protein groups
Filter for e.g. contaminants
Peptides ≥ 2 in at least 3/4 n per group
2415 protein groups
Filter against background over FLAG lines
by t-test p <0.05, FDR <0.05, log2fc >1
570 protein groups
Comparison TECPR2 WT vs MUT with TMEM192-HA
by t-test p <0.05, FDR <0.05, log2fc >1 (class 1)
by t-test p <0.05, FDR <0.05, log2fc >0.1 (class 2)
c
TMEM192 -HA
TMEM192 -FLAG
TECPR2
WT ≥0.95 ≥0.97
MUT ≥0.96 ≥0.96
Minimum r of 4 n, LFQ intensities
d
Proteins enriched over FLAG cells
TECPR2 WT
TECPR2 MUT
112
366
92
total: 570 proteins
e
Autophagy
Cell-cell adhesion
COPII vesicle coating
Endosome to Golgi transport
Endosome to lysosome transport
ER to Golgi transport
Golgi to ER transport
Intracellular protein transport
Lysosomal lumen acidification
Lysosome localization
Lysosome organization
Multivesicular body assembly
Autophagosome
Cytosol
Early endosome
ER
ERGIC
Extracellular exosome
Golgi apparatus
Late endosome
Lysosomal lumen
Lysosomal membrane
Lysosome
Plasma membrane
Vesicle
BP
CC
ns <0.05 <E-02 <E-10 <E-20 <E-30
FDR
1
57
f
Lysosomal content
Autophagosome content*
515
55
235
24
log2 fold-change
2
1
0
-1
-2
*Zellner et al. 2021 3
Up in TECPR2 MUT
Down in TECPR2 MUT
g
kDa
WT
MUT
TECPR2
75
100
63
245
135
63
BiP
Calnexin
Calreticulin
Giantin
GM130
Tubulin
h
ssRFP-GFP-KDEL reporter
mKeima-RAMP4 reporter
Averaged red/green ratio
1.0
0.8
0.6
0.4
0.2
0.0
0
8
16
24
32
40
48
56
Time (hours)
WT DMSO
WT Torin1
MUT DMSO
MUT Torin1

Supplementary Figure 7: **Analysis of lysosome composition and ER-phagy.**

a Homogenates from 293T cells expressing C-terminally 3xHA or 2xFLAG-tagged TMEM192 were subjected to HA-IP followed by immunoblot analysis. LAMP1 and NPC1 served as lysosomal controls, calnexin, GM130 and TOMM40 as organelle markers. **b** Schematic representation of Lyso-IP proteomic data filtering and statistical processing. **c** Pearson correlation of four biological replicates based on LFQ intensities of proteins identified by LysoIP proteomics. **d** Venn graph of proteins enriched in TMEM192-3HA expressing cells compared to FLAG background. **e** GO term enrichment of proteins enriched in TMEM192-3HA expressing cells. Dot size correlates to number of proteins, dot color to term enrichment (FDR). **f** Comparative analysis of lysosomal proteins found in this study and previously mapped autophagosome content [3]. Brown circles indicate commonly altered proteins for which $\log_2$ fold changes upon expression of mutant TECPR2 are depicted as bar graph. **g** Lysates from TECPR2 WT and MUT 293T cells were analyzed by immunoblotting. PCNA served as loading control. **h** TECPR2 WT and MUT cells expressing ssRFP-GFP-KDEL or mKeima-RAMP4 reporter were seeded in 384-well plates in the presence of Doxycycline for 24 hours (KDEL) or 16 h without additional induction (RAMP4). Cells were subsequently treated with DMSO or 250 nM Torin1. Red and green signals were measured over a total period of 60 hrs using the IncuCyteS3. ER-phagy flux represented by the RFP/GFP (red/green) ratio (total fluorescent intensities). Data representative for two biological replicates, measured in three technical replicates each. Line graphs depict averaged values. Source data are provided in Source Data file.

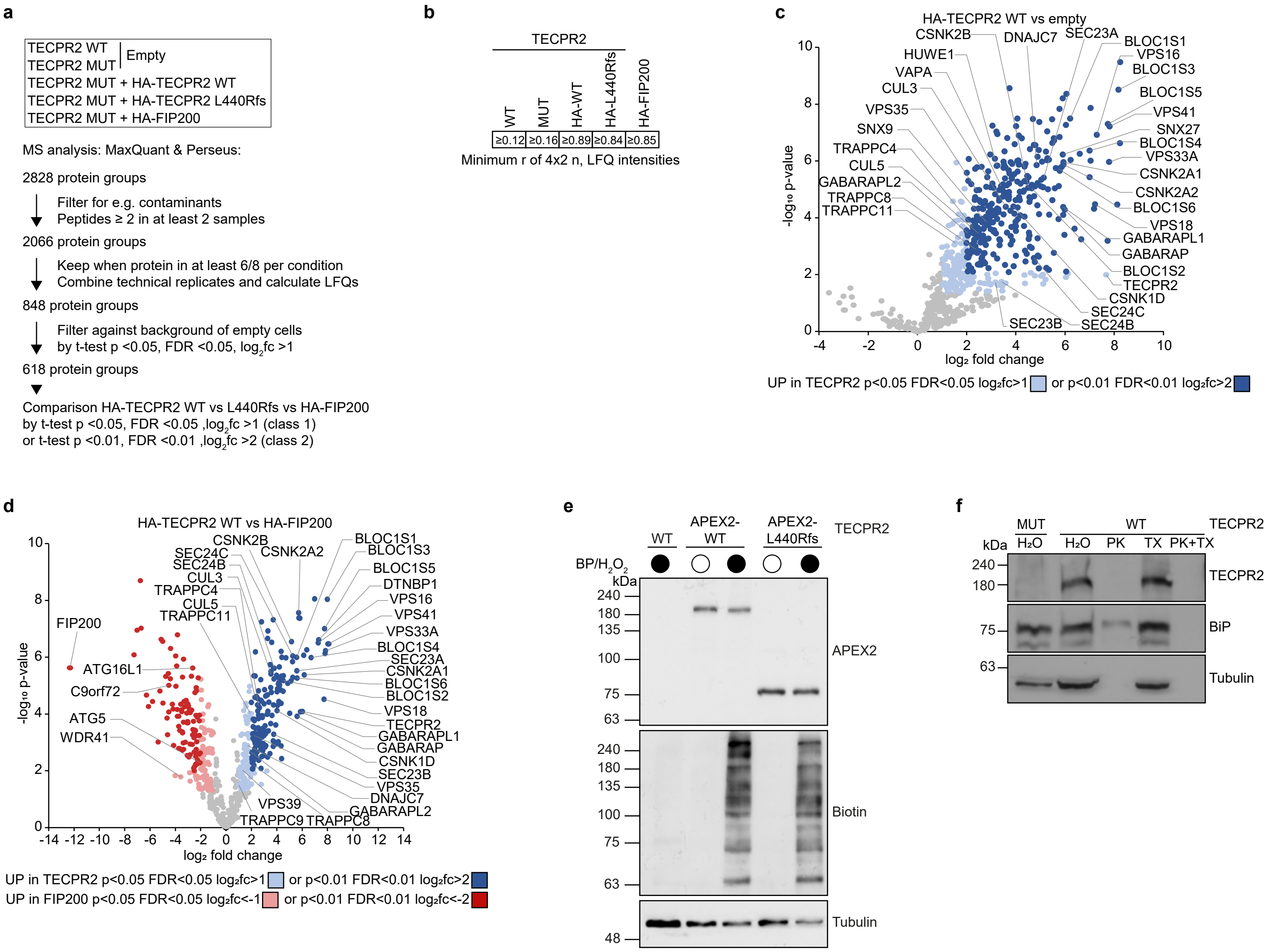
a
TECPR2 WT
TECPR2 MUT
Empty
TECPR2 MUT + HA-TECPR2 WT
TECPR2 MUT + HA-TECPR2 L440Rfs
TECPR2 MUT + HA-FIP200
MS analysis: MaxQuant & Perseus:
2828 protein groups
Filter for e.g. contaminants
Peptides ≥ 2 in at least 2 samples
2066 protein groups
Keep when protein in at least 6/8 per condition
Combine technical replicates and calculate LFQs
848 protein groups
Filter against background of empty cells
by t-test p <0.05, FDR <0.05, log2fc >1
618 protein groups
Comparison HA-TECPR2 WT vs L440Rfs vs HA-FIP200
by t-test p <0.05, FDR <0.05 ,log2fc >1 (class 1)
or t-test p <0.01, FDR <0.01 ,log2fc >2 (class 2)
b
TECPR2
WT
MUT
HA-WT
HA-L440Rfs
HA-FIP200
≥0.12
≥0.16
≥0.89
≥0.84
≥0.85
Minimum r of 4x2 n, LFQ intensities
c
HA-TECPR2 WT vs empty
-log₁₀ p-value
log₂ fold change
CSNK2B
DNAJC7
SEC23A
BLOC1S1
VPS16
BLOC1S3
HUWE1
VAPA
CUL3
BLOC1S5
VPS35
VPS41
SNX9
SNX27
BLOC1S4
VPS33A
TRAPPC4
CUL5
CSNK2A1
GABARAPL2
CSNK2A2
TRAPPC8
BLOC1S6
TRAPPC11
VPS18
GABARAPL1
GABARAP
BLOC1S2
TECPR2
CSNK1D
SEC24C
SEC23B
SEC24B
UP in TECPR2 p<0.05 FDR<0.05 log₂fc>1
or p<0.01 FDR<0.01 log₂fc>2
d
HA-TECPR2 WT vs HA-FIP200
-log₁₀ p-value
log₂ fold change
CSNK2B
BLOC1S1
SEC24C
CSNK2A2
BLOC1S3
SEC24B
BLOC1S5
CUL3
DTNBP1
TRAPPC4
VPS16
CUL5
VPS41
TRAPPC11
FIP200
VPS33A
BLOC1S4
SEC23A
ATG16L1
CSNK2A1
BLOC1S6
C9orf72
BLOC1S2
VPS18
ATG5
TECPR2
GABARAPL1
WDR41
GABARAP
CSNK1D
SEC23B
VPS35
DNAJC7
VPS39
GABARAPL2
TRAPPC9
TRAPPC8
UP in TECPR2 p<0.05 FDR<0.05 log₂fc>1
or p<0.01 FDR<0.01 log₂fc>2
UP in FIP200 p<0.05 FDR<0.05 log₂fc<-1
or p<0.01 FDR<0.01 log₂fc<-2
e
WT
APEX2-WT
APEX2-L440Rfs
TECPR2
BP/H2O2
kDa
240
180
135
100
75
63
48
APEX2
Biotin
Tubulin
f
MUT
WT
TECPR2
kDa
H₂O
H₂O
PK
TX
PK+TX
240
180
75
63
TECPR2
BiP
Tubulin

Supplementary Figure 8: **TECPR2 interactome analysis.**

a Schematic representation of HA-IP proteomic data filtering and statistical processing. **b** Pearson correlation of 4 biological replicates based on LFQ intensities of identified proteins. **c, d** Volcano plot of candidate interacting proteins of HA-TECPR2 WT expressed in TECPR2 MUT cells compared to empty TECPR2 MUT cells (**c**) or HA-FIP200 expressed in TECPR2 MUT cells (**d**) identified by HA-IP proteomics. Enriched proteins were highlighted in light (p <0.05, FDR <0.05 and log2 fold change >1, two-sided t-test) or dark blue (p <0.01, FDR <0.01 and log2 fold change >2, two-sided t-test, n=4 independent experiments). **e** APEX2-TECPR2 WT or L440Rfs expressing 293T cells were labeled with biotin, lysed and analyzed by immunoblotting. Tubulin served as loading control. **f** Immunoblots of homogenates from TECPR2 WT and MUT cells left untreated (H2O) or incubated with proteinase K (PK), Triton-X100 (TX) or both. BiP and Tubulin served as controls. Source data are provided in Source Data file.

Supplementary References:

1 Leidal, A. M. *et al.* The LC3-conjugation machinery specifies the loading of RNA-binding proteins into extracellular vesicles. *Nat Cell Biol* **22**, 187-199, doi:10.1038/s41556-019-0450-y (2020).

2 Kugeratski, F. G. *et al.* Quantitative proteomics identifies the core proteome of exosomes with syntenin-1 as the highest abundant protein and a putative universal biomarker. *Nat Cell Biol* **23**, 631-641, doi:10.1038/s41556-021-00693-y (2021).

3 Zellner, S., Schifferer, M. & Behrends, C. Systematically defining selective autophagy receptor-specific cargo using autophagosome content profiling. *Mol Cell* **81**, 1337-1354 e1338, doi:10.1016/j.molcel.2021.01.009 (2021).
