# Supplementary material for: Spatial proteomics reveals secretory pathway disturbances caused by neuropathy-associated TECPR2
Source: Nat Commun. 2023 Feb 16;14:870. doi: 10.1038/s41467-023-36553-6 (PMC9935918; doi:10.1038/s41467-023-36553-6)
Supplement: Supplementary file 3 — Description of Additional Supplementary Files [file 41467_2023_36553_MOESM3_ESM.docx]

**Description of Additional Supplementary Files**

File Name: Supplementary Data 1 - 8

Description: **Summary of proteomic data sets.**

An inventory of all datasets across the different proteomics experiments is followed by detailed information on each dataset including label-free quantification and statistical analysis.

File Name: Supplementary Data 9

Description: **List of Resources.**

Summary of all used reagents and tools including unique identifiers, order numbers, version information and other additional information.
