# Supplementary material for: Spatial proteomics reveals secretory pathway disturbances caused by neuropathy-associated TECPR2
Source: Nat Commun. 2023 Feb 16;14:870. doi: 10.1038/s41467-023-36553-6 (PMC9935918; doi:10.1038/s41467-023-36553-6)
Supplement: Supplementary file 6 — Reporting Summary [file 41467_2023_36553_MOESM6_ESM.pdf]

nature portfolio

Corresponding author(s): Christian Behrends

Last updated by author(s): Jan 15, 2023

Reporting Summary

Nature Portfolio wishes to improve the reproducibility of the work that we publish. This form provides structure for consistency and transparency in reporting. For further information on Nature Portfolio policies, see our Editorial Policies and the Editorial Policy Checklist.

Statistics

For all statistical analyses, confirm that the following items are present in the figure legend, table legend, main text, or Methods section.

n/a	Confirmed	
☐	☒	The exact sample size (*n*) for each experimental group/condition, given as a discrete number and unit of measurement
☐	☒	A statement on whether measurements were taken from distinct samples or whether the same sample was measured repeatedly
☐	☒	The statistical test(s) used AND whether they are one- or two-sided<br>*Only common tests should be described solely by name; describe more complex techniques in the Methods section.*
☒	☐	A description of all covariates tested
☒	☐	A description of any assumptions or corrections, such as tests of normality and adjustment for multiple comparisons
☐	☒	A full description of the statistical parameters including central tendency (e.g. means) or other basic estimates (e.g. regression coefficient) AND variation (e.g. standard deviation) or associated estimates of uncertainty (e.g. confidence intervals)
☐	☒	For null hypothesis testing, the test statistic (e.g. *F*, *t*, *r*) with confidence intervals, effect sizes, degrees of freedom and *P* value noted<br>*Give P values as exact values whenever suitable.*
☒	☐	For Bayesian analysis, information on the choice of priors and Markov chain Monte Carlo settings
☒	☐	For hierarchical and complex designs, identification of the appropriate level for tests and full reporting of outcomes
☐	☒	Estimates of effect sizes (e.g. Cohen's *d*, Pearson's *r*), indicating how they were calculated

Our web collection on statistics for biologists contains articles on many of the points above.

Software and code

Policy information about availability of computer code

Data collection	Immunoflourescence images were either taken on a Zeiss LSM800 microscope with an oil-immersion 60x objective (Zeiss) or on an OPERA LX high-content screening microscope (Perkin Elmer). Mass spectrometry data were acquired using an Easy-nLC1200 HPLC (Thermo Scientific) coupled to an Q Exactive HF mass spectrometer via nanospray ionization (Thermo Scientific). The LC-MS system was controlled by Xcalibur (Thermo Scientific, version 4.3). Electron microscopy images were taken on a JEM-1400+ (JEOL) equipped with a XF416 (TVIPS) and the EM-Menu software (TVIPS). ER-phagy experiments were performed using an IncuCyte 53 (Sartorius) and SH800S Cell Sorter (Sony).
Data analysis	Immunofluorescence data were analyzed using ImageJ/Fiji (version 1.8.0), Zenblue 2011 (Zeiss, verion 2.5) or Columbus (Perkin Elmer, version 2.9.0). Proteomic raw data were processed using MaxQuant (version 1.6.0.1) and further analyzed using Perseus (version 1.6.5.0) or online tools such as DAVID (LHRI, version 2021) and BioGrid (version 3.5.187). Electron microscopy data were generated using TEMCenter software (JEOL, version 1.7.3.1527) and ShotMeister software (JEOL, version 2.3.15.5). Immunoblotting images were assembled and general figures generated in Adobe Photoshop (version 26.31).

For manuscripts utilizing custom algorithms or software that are central to the research but not yet described in published literature, software must be made available to editors and reviewers. We strongly encourage code deposition in a community repository (e.g. GitHub). See the Nature Portfolio guidelines for submitting code & software for further information.

Data

Policy information about availability of data

All manuscripts must include a data availability statement. This statement should provide the following information, where applicable:

- Accession codes, unique identifiers, or web links for publicly available datasets
- A description of any restrictions on data availability
- For clinical datasets or third party data, please ensure that the statement adheres to our policy

All data are available upon reasonable request. All mass spectrometry / proteomic datasets reported in this study have been deposited to the ProteomeXchange Consortium via the PRIDE repository and are publicly available via the accession number PXD031874 [http://www.ebi.ac.uk/pride/archive/projects/PXD031874]. A human reference proteome (Uniprot-FASTA, UP000005640, downloaded September 2017) was used for protein identification. Source data / supplementary information are provided with this publication.

Human research participants

Policy information about studies involving human research participants and Sex and Gender in Research.

Reporting on sex and gender	No human research participants.
Population characteristics	No human research participants.
Recruitment	No human research participants.
Ethics oversight	No human research participants.

Note that full information on the approval of the study protocol must also be provided in the manuscript.

Field-specific reporting

Please select the one below that is the best fit for your research. If you are not sure, read the appropriate sections before making your selection.

[x] Life sciences [ ] Behavioural & social sciences [ ] Ecological, evolutionary & environmental sciences

For a reference copy of the document with all sections, see nature.com/documents/nr-reporting-summary-flat.pdf

Life sciences study design

All studies must disclose on these points even when the disclosure is negative.

Sample size	No sample size calculation was performed prior to experiments, sample sizes were based on commonly used sample sizes instead. For proteomic analyses, sample size was 4, for all other quantifications at least 3, and included appropriate control samples. The precise number of replicates are described in the respective figure legend.
Data exclusions	No data were excluded.
Replication	All reported data were reproducible and similar effects were multiple times (at least in triplicates). For some experiments (e.g. immunofluorescence analysis) quantification was provided, including the exact number of replicates.
Randomization	Samples were not randomized, as it was either not applicable or not necessary due to automated and unbiased analysis (e.g. quantification of high-throuput images). All experiments included appropriate controls in the same cell system that were processed and analyses identically.
Blinding	Blinding was not relevant to our study. All experiments were performed by the same investigator in an objective manner. Quantification of proteomic and immunoflourescence data was performed identically for all compared samples and controls.

Reporting for specific materials, systems and methods

We require information from authors about some types of materials, experimental systems and methods used in many studies. Here, indicate whether each material, system or method listed is relevant to your study. If you are not sure if a list item applies to your research, read the appropriate section before selecting a response.

Materials & experimental systems

n/a	Involved in the study
	☒ Antibodies
	☒ Eukaryotic cell lines
☒	Palaeontology and archaeology
☒	Animals and other organisms
☒	Clinical data
☒	Dual use research of concern

Methods

n/a	Involved in the study
☒	ChIP-seq
☒	Flow cytometry
☒	MRI-based neuroimaging

Antibodies

Antibodies used

All primary antibodies used in this study are listed below and were used at a concentration of 1:1000 (immunoblotting) or 1:300-500 (immunostaining) unless otherwise specified:
ADAMTS1 Abcam Cat#ab39194; RRID:AB_2221876
APEX2 (IgG2A) Regina Feederle, HZM Custom made
ATP1A1 Abcam Cat#ab7671; RRID:AB_306023
B4GALT3 Proteintech Cat#11041-1-AP; RRID:AB_2290072
BAG2 Biomol Cat#A304-751A; RRID:AB_2620946
beta-ACTIN Sigma Cat#A1978; RRID:AB_476692
BIOTIN Pierce Cat#31852; RRID:AB_228243
BiP Cell Signaling Cat#3177; RRID:AB_2119845
CALNEXIN Abcam Cat#ab22595; RRID:AB_2069006
Calreticulin santa cruz Cat#sc-6467; RRID:AB_667958
CD63 abcam Cat#ab59479; RRID:AB_940915
CLPTM1L Sigma Cat#HPA014791; RRID:AB_1847250
c-myc Bethyl Cat#A190-104A; RRID:AB_66864
CRLF1 Novus Cat# NBP1-85606; RRID:AB_11011347
DTNBP1 Bethyl Cat#A303-360A; RRID:AB_10951943
EDIL3 Abcam Cat#ab190692; RRID:-
ERGIC1 Proteintech Cat#16108-1-AP; RRID:AB_2098435
ERGIC53 Santa Cruz Cat#sc-398777; RRID:-
EXTL2 Abcam Cat#ab168391; RRID:-
Flag M2 Cell Signaling Cat#2368; RRID:AB_2217020
GABARAP Abcam Cat#109364; RRID:AB_10861928
Giantin Biolegend Cat#924302;RRID:AB_2565451
GM130 Abcam Cat# ab52649; RRID:AB_880266
GOLIM4 Abcam Cat# ab28049; RRID:AB_732692
HA.11 Clone 16B2 Covance/Biolegend Cat#MMS-101P, Cat#901501; RRID:AB_2314672, RRID:AB_2565006
HS6ST2 Abcam Cat# ab122220; RRID:AB_11132121
LAMP1 (IF 1:100) Abcam/DSHB Cat#ab24170, H4A3; RRID:AB_775978, RRID:AB_2296838
LAMP2 (IF 1:100) Abcam Cat#ab25631; RRID:AB_470709
LAMTOR1 Cell Signaling Cat# 8975S; RRID:AB_10860252
LAMTOR2 Cell Signaling Cat#8145; RRID:AB_10971636
LAMTOR3 Cell Signaling Cat#8168; RRID:AB_10949501
LC3B Cell Signaling Cat#2775S; RRID:AB_915950
LGALS3BP Proteintech Cat#10281-1-AP; RRID:AB_2137066
LRP1 Abcam Cat#ab92544; RRID:AB_2234877
M6PR Abcam Cat#ab2733; RRID:AB_2122792
MTOR Cell Signaling Cat#2983; RRID:AB_2105622
myc 9E10 Regina Feederle, HZM Custom made
NCAM1 (WB 1:500, IF 1:100) Merck Cat#AB5032; RRID:AB_2291692
NEK9 Abcam Cat#ab138488; RRID:-
NID1 Invitrogen Cat#PA5-99666; RRID:AB_2818599
NPC1 Abcam Cat#ab134113; RRID:AB_2734695
PCNA Santa Cruz Cat#sc-7907; RRID:AB_2160375
PLXDC2 Novus Cat#NBP1-76858; RRID:AB_11014794
PLXNA1 R&D Cat#AF4309; RRID:AB_10645644
PLXNA2 Abcam Cat#ab39357; RRID:AB_1142286
RAB5C Sigma Cat#HPA003426; RRID:AB_1079735
SEC12 Novus Cat#NBP1-87056; RRID:AB_11014112
SEC13 Novus Cat#AF9055-100; RRID:-
SEC24C Abcam Cat#ab122633; RRID:AB_11130089
SEC24D Cell Signaling Cat#14687; RRID:AB_2798574
SEC31A BD Cat#612351; RRID:AB_399717
SLC38A9 Abcam Cat#ab81687; RRID:AB_1860451

	SPG20 Proteintech Cat#13791-1-AP; RRID:AB_2195683<br>TECPR2 AG Behrends Custom made<br>TNC Abcam Cat#ab108930; RRID:AB_10865908<br>TOLLIP Abcam Cat#ab187198; RRID:-<br>TOMM40 Abcam Cat#ab185543; RRID:-<br>TRAPPC11 Sigma Cat#HPA045427; RRID:-<br>TRAPPC8 Sigma Cat# HPA041107; RRID:AB_10793869<br>TRAPPC9 Proteintech Cat#16014-1-AP; RRID:AB_2256482<br>TSG101 Abcam Cat#ab30871; RRID:AB_2208084<br>TUBULIN Abcam Cat# ab7291; RRID:AB_2241126<br>VAPA Sigma Cat# HPA009174; RRID:AB_1080549<br>VAPB Sigma Cat# HPA013144; RRID:AB_1858717<br>VCP Bethyl Cat#A300-588A; RRID:AB_495511<br>VPS11 Abcam Cat#ab125083; RRID:AB_10975464<br><br>Secondary antibodies were used at a concentration of 1:10,000 (immunoblotting) or 1:600 (immunofluorescence):<br>anti-goat-HRP Promega Cat#V8051; RRID:AB_ 430838<br>anti-mouse-HRP Promega Cat#W402B,RRID:AB_ 430834<br>anti-rabbit-HRP Promega Cat#W4011,RRID:AB_ 430833<br>anti-rat-HRP Sigma Cat#A-9037;RRID:AB_258429<br>Donkey anti-goat-488 Life Technologies Cat#A11055;<br>Donkey anti-goat-488 Life Technologies Cat#A11055;RRID:AB_2534102<br>Donkey anti-mouse-488 Life Technologies Cat#A21202;RRID:AB_141607<br>Donkey anti-rabbit-488 Life Technologies Cat#A21206;RRID:AB_2535792<br>Goat anti-rabbit-488 Life Technologies Cat#A11034;RRID:AB_2576217<br>Goat anti-mouse-488 Life Technologies Cat#A11001;RRID:AB_2534069<br>Donkey anti-goat-555 Life Technologies Cat#A21432;RRID:AB_2535853<br>Donkey anti-mouse-555 Life Technologies Cat#A32773;RRID:AB_2762848<br>Donkey anti-rabbit-555 Life Technologies Cat#A31572;RRID:AB_162543<br>Goat anti-mouse-555 Life Technologies Cat#A21424;RRID:AB_141780<br>Donkey anti-goat-640 Life Technologies Cat#A32849;RRID:AB_2762840
Validation	Validation of all commercially available antibodies can be found on the respective manufacturer's website/manual and in general included immunoblotting or immunofluorescence stainings. Self-genereated TECPR2, APEX2 and MYC antibodies were validated in previously published studies (Stadel et al. 2015, Fraiberg et al. 2021, Zellner et al. 2021). All used primary antibodies were additionally confirmed in our lab for proper protein detection via immunoblotting or immunofluorescence.

Eukaryotic cell lines

Policy information about cell lines and Sex and Gender in Research

Cell line source(s)	All cell lines were purchased from ATCC (293T cells, Cat#CRL-3216; Hela cells, Cat#CCL-2; SH-SY5Y, Cat#CRL-2266) or received from Q-State Biosciences or the Elazar lab.
Authentication	All cell lines were either derived from ATCC authenticated cell lines or tested by the respective lab that generated these lines (e.g. by sequencing, immunoblotting and immunofluorescence).
Mycoplasma contamination	All cell lines were tested negative for mycoplasma contaminations.
Commonly misidentified lines<br>(See ICLAC register)	No commonly misidentified cell lines were used in this study.
